# Cage and maternal effects on the bacterial communities of the murine gut

**DOI:** 10.1038/s41598-021-89185-5

**Published:** 2021-05-10

**Authors:** Gurdeep Singh, Andrew Brass, Sheena M. Cruickshank, Christopher G. Knight

**Affiliations:** 1 Faculty of Biology, Medicine and Health, Lydia Becker Institute of Immunology and Inflammation, Manchester Academic Health Science Centre, A.V. Hill Building, The University of Manchester, Oxford Road, Manchester, M13 9PT UK; 2 Faculty of Biology, Medicine and Health, Division of Informatics, Imaging and Data Sciences, Stopford Building, The University of Manchester, Oxford Road, Manchester, M13 9PT UK; 3 Faculty of Science and Engineering, School of Earth and Environmental Sciences, Michael Smith Building, The University of Manchester, Oxford Road, Manchester, M13 9PT UK

**Keywords:** Computational biology and bioinformatics, Immunology, Microbiology

## Abstract

Findings from gut microbiome studies are strongly influenced by both experimental and analytical factors that can unintentionally bias their interpretation. Environment is also critical. Both co-housing and maternal effects are expected to affect microbiomes and have the potential to confound other manipulated factors, such as genetics. We therefore analysed microbiome data from a mouse experiment using littermate controls and tested differences among genotypes (wildtype versus colitis prone-*mdr1a*^*−/−*^), gut niches (stool versus mucus), host ages (6 versus 18 weeks), social groups (co-housed siblings of different genotypes) and maternal influence. We constructed a 16S phylogenetic tree from bacterial communities, fitting random forest models using all 428,234 clades identified. Models discriminated all criteria *except* host genotype, where no community differences were found*.* Host social groups differed in abundant, low-level, taxa whereas intermediate phylogenetic and abundance scales distinguished ages and niches. Thus, a carefully controlled experiment treating evolutionary clades of microbes equivalently without reference to taxonomy, clearly identifies whether and how gut microbial communities are distinct across ecologically important factors (niche and host age) and other experimental factors, notably cage effects and maternal influence. These findings highlight the importance of considering such environmental factors in future microbiome studies.

## Introduction

One of the key biomedical discoveries of recent years has been the critical role of the microbiome in host function and disease. The microbiome is implicated in a huge range of functions in health, such as gut barrier and immune function, and disease, including obesity, autoimmune disease and allergy^1^. While associations of the microbiome with human health and disease multiply, questions of causality become increasingly important. Unpicking that causality requires careful experiments, which are typically not feasible in humans. Therefore the science of the microbiome has been, and is being, built on animal research, primarily in mice, with an exponential rise in such publications.

**Figure 1:**
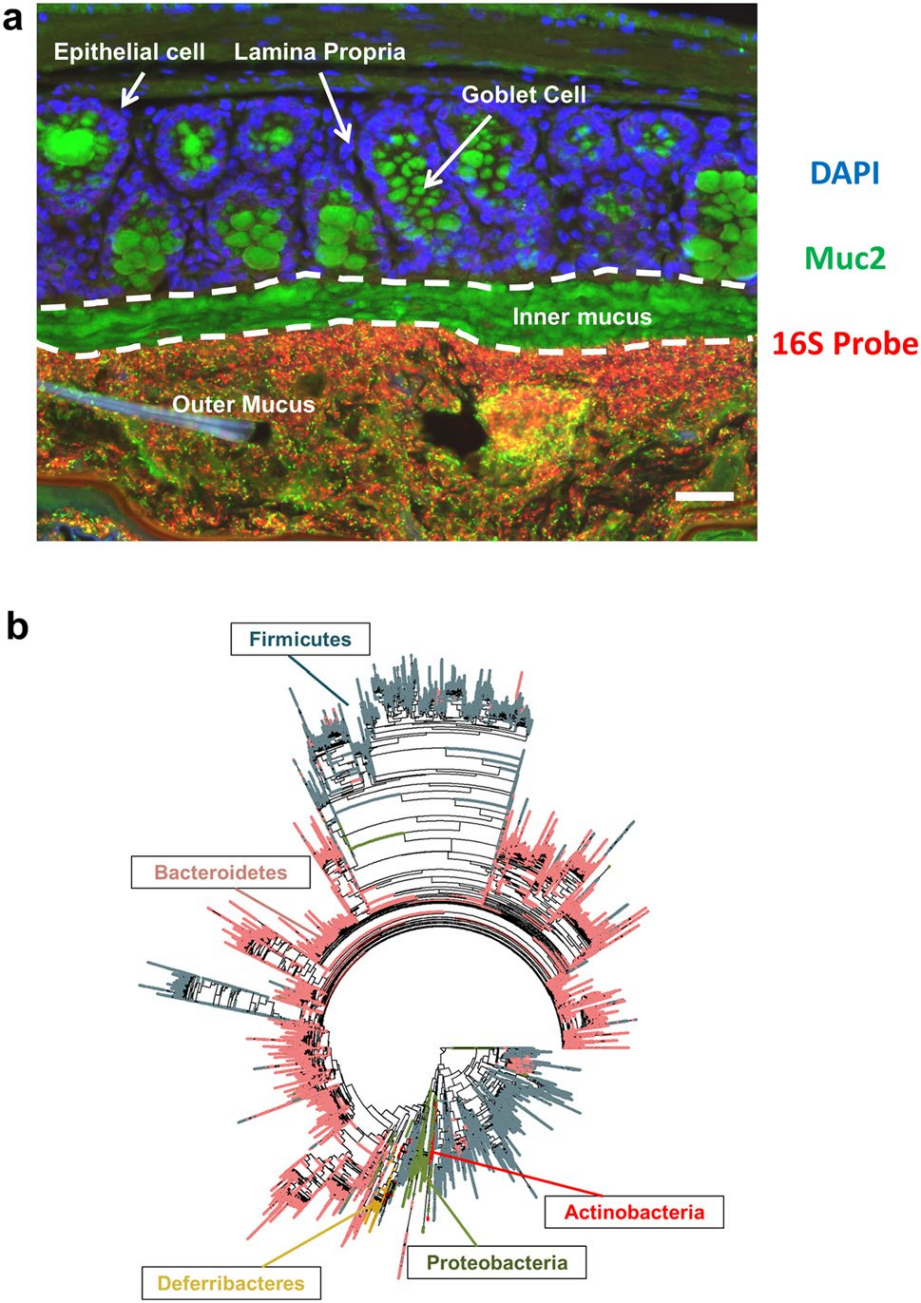
Distribution of phyla across the phylogenetic tree. Colonic tissue sections from a male wildtype (WT) mouse was stained with a fluorescent DNA probe specific for the 16S rRNA gene to identify bacteria (red), a Muc2 antibody (green) to identify mucus and counterstained with DAPI (blue) (**a**). A phylogenetic tree of 16S rRNA sequences derived from the gut microbiota of FVB wildtype (WT) mice and *mdr1a*^*−/−*^ mice (**b**). The distribution of major gut phyla are highlighted on the tree: Firmicutes (grey), Bacteroidetes (pink), Proteobacteria (olive), Actinobacteria (red), and Deferribacteres (gold). The same tree is shown, coloured by other criteria, in Supplementary Figure S1. Figure (**b**) was produced in R 3.6.0 for Windows.

Unfortunately, microbiome studies can be inaccurate or biased due to both experimental and analytical factors^2^. Notably, experimental design issues, such as the sampling site of the microbiota^3^ and the environment of the host^4^, can have a large impact. For instance, gut microbiota research has tended to focus on stool samples, where changes in the stool microbiota have been associated with several diseases, most notably inflammatory bowel disease (IBD)^5,6^. However, stool samples alone do not fully reflect the total gut microbiota. Bacteria inhabit various niches along the length of the gut, particularly the mucus layer overlaying the intestinal epithelial cells^3,7^ (Fig. 1a). We have also shown that these bacterial niche populations can be impacted independently of effects within the stools^7^. Furthermore, mice^8^, like humans^9^, share gut microbes among co-housed individuals which, in mice, is reinforced by coprophagy. Thus, individual mice in the same cage have similar microbial communities and any differences among the microbial communities of different cages can dominate analysis^10^. Surprisingly, few studies report on how mice are caged and whether they are littermates^11,12^. Intergenerational differences in the murine microbiota has also been reported^13^. Not reporting these critical factors leads to questions about the reproducibility of the research, as discussed by Stappenbeck and Virgin^10^. Indeed, there is the possibility that any apparent changes in the microbiome that arise between mouse groups are mistakenly assigned as treatment effects. It is therefore important to define more clearly the contribution of host environment in animal microbiome studies and develop robust tools that interrogate this.

Some of the most common methods of microbiome analysis also have the potential to obscure findings. Bacteria interact in complex communities. Thus, the power of individual statistics, either ones that focus on large scale shifts among phyla (e.g. the Firmicutes to Bacteroidetes ratio^14^) or that single out particular species differences (e.g. *Lactobacillus reuteri* enrichment in obesity^15^), potentially miss functional impacts. Such narrow foci may lead to bias and over-interpretation of the relative importance of single species changes. To date, even though microbiome studies frequently repeat analyses at multiple taxonomic levels and tools have been developed capable of highlighting specific important taxa within a tree (e.g.^16^), it is rare to examine all taxa across a phylogenetic tree in a single analytical framework, enabling the relative importance of community differences at different phylogenetic scales to be assessed.

Here we ask whether some of the experimental features that may confound analyses—cage and maternal effects, have similar or different effects on microbial communities to experimental variables of interest (niche within the murine gut, mouse age and genotype of the host). We use 16S rDNA data from a carefully controlled experiment, with wild type and colitis-prone *mdr1a*^*−/−*^ mice (where some differences have been reported in the bacterial community of the gut mucus^17^), in co-housed, mixed genotype cages. To minimise issues of potential inaccuracy and bias, we do not define operational taxonomic unit (OTUs). Instead, we use the sequences themselves to estimate the phylogenetic relationships amongst organisms and thereby abundances at different phylogenetic scales. Only after analysis do we draw on a wider understanding of microbial taxonomy to interpret the findings. We find striking cage and maternal effects on the microbial communities, which are clearly distinguished among niches and host age, highlighting the importance of factoring housing into experimental design and analysis.

## Materials and methods

### Animal maintenance

The breeding and sampling design is detailed in Supplementary Figure S1. *Mdr1a*^*−/−*^ mice (FVB.129P2-Abcb1atm1Bor N7)^18^ were bred with control FVB mice purchased from Taconic Biosciences (Albany, NY), to produce the F2 generation. Heterozygous parents were then used to give rise to experimental mice, allowing litters to contain a mix of genotypes. Experimental male mice from each litter were co-housed in individually vented cages, in the same room and on the same rack in the animal facility. Thus, WT and *mdr1a*^*−/−*^ mice from the same litters were used for all subsequent experiments, in mixed genotype cages. Male mice at 6 and 18 weeks of age were used for experiments. Different mice were sampled at each time point. All mice received the same food (Beekay Rat and Mouse Diet No1 pellets; B&K Universal, UK) and irradiated water which were available ad libitum*,* prior to and during the experiment. Ambient temperature was maintained at 21 (± 2 °C) and the relative humidity was 55 (± 10%) with a 12 h light/dark cycle. All animals were kept under specific, pathogen-free (SPF) conditions at the University of Manchester, where cage-cross contamination was prevented at every stage via strict hygiene procedures. Experiments were performed under a project license approved by the institutional Animal Welfare and Ethical Review Body (GRENCIS 70/8127) and according to the regulations issued by the Home Office under amended ASPA, 2012.

### Isolation of genomic DNA

Sample collection and processing was performed as described by Glymenaki et al.^17^. In brief, samples were harvested from mice at two time points, 6 and 18 weeks of age. Stool samples were collected from mice in individual autoclaved cages into sterile tubes and snap frozen on dry ice. Mice were sacrificed via CO_2_ inhalation, the proximal colon was cut open and the colonic mucus scraped using cell scrapers and Inhibitex buffer (QIAGEN, Manchester, UK) and snap frozen until use. Genomic DNA was extracted using QIAamp Fast Stool Mini-Kits according to the manufacturer’s instructions (QIAGEN).

### Histology

Snips of the proximal colon were fixed in Carnoy’s solution (60% methanol, 30% chloroform, 10% glacial acetic acid), incubated in two changes of dry methanol (Sigma-Aldrich, Dorset, UK) for 30 min each, followed by absolute ethanol (ThermoFisher Scientific, Paisley, UK) for two incubations at 30 min each. Finally, tissue cassettes were processed in a Micro-spin Tissue Processor STP120 (ThermoFisher Scientific) and immersed in paraffin. Colon snips were embedded in paraffin blocks using a Leica Biosystems embedding station (Leica Biosystems, Milton Keynes, UK), with the luminal surface of the colon exposed for tissue sectioning. 5 µm tissue sections were cut using a Leica Biosystems microtome and adhered to uncoated microscope slides (ThermoFisher Scientific). Slides were dried for 48 h at 50 °C before use. Histological analysis was used to determine that all five of the 18 week-old *mdr1a*^*−/−*^ mice had indications of moderate or mild colitis, with a loss of healthy gut architecture^17^.

### Fluoresence in situ hybridisation (FISH)

FISH was performed as described previously^17^. In brief, FISH staining was performed using the universal bacterial probe-EUB338 (5′-Cy3-GCTGCCTCCCGTAGGAGT-3′), followed by immunostaining with a rabbit polyclonal MUC2 antibody and goat anti-rabbit Alexa-Fluor 488 antibody (Life Technologies, Paisley, UK). Slides were imaged using a BX51 upright microscope and a Coolsnap EZ camera (OLYMPUS, Tokyo, Japan) and images were processed using Image J^19^.

### 16S rRNA gene sequencing processing

16S amplicon sequencing targeting the V3 and V4 variable regions of the 16S rRNA (341F: 5′-TCGTCGGCAGCGTCAGATGTGTATAAGAGACAGCCTACGGGNGGCWGCAG-3′ and 805R: 5′-GTCTCGTGGGCTCGGAGATGTGTATAAGAGACAGGACTACHVGGGTATCTAATCC-3′) was performed on the Illumina MiSeq platform (Illumina, California, USA) according to manufacturer’s guidelines and generated paired-end reads of 300 bp in each direction. DNA from all samples was extracted using the same extraction kit. However, they were sequenced across different runs, with technical replicate samples sequenced multiple times as an internal control between each run. Illumina reads were demultiplexed to remove adapter sequences and trim primers. Illumina paired-end reads were merged together using SeqPrep^20^ and submitted to MG-RAST’s metagenomics pipeline^21^. Reads were pre-processed to remove low-quality and uninformative reads using SolexQA^22^. The quality-filtering process included removal of reads with low quality ends (i.e. ambiguous leading/trailing bases) and the removal of reads with a read length two standard deviations below the mean. Artificial duplicate reads were then removed based on MG-RAST’s pipeline.

The resulting FASTQ files for every sample were merged into a single file of 590,822 sequences to simplify processing, manually adding 3 known Archaeal 16S rRNA sequences from *Acidilobus saccharovorans*, *Sulfolobus tokodaii* and *Methanobrevibacter smithii*. Sequences were aligned using a specialist 16S RNA aligner using the Infernal algorithm^23^, via a web-based interface provided by the Ribosomal Database Project^24^. This file was then manually curated in R^25^. In brief, we determined the first and last position of each base for every sequence. 92% of all sequences started around position 710. 87% of sequences had their last base around position 2800. We therefore trimmed all positions before 710 and after 2800 to remove unnecessary spaces introduced by the aligner. The number of aligned bases in each sequence was then recorded and the distribution of continuously aligned bases was examined. The large majority of sequences (~ 84%) had > 437 bases and so any sequence that had less than 437 continuously aligned bases was discarded. The remaining 496,550 sequences were taken forward for analysis. All sequences were identified using BLAST+ and the top hit for each sequence was recorded^26^. The ‘classification’ function in the ‘taxize’ R package^27^ was then used to assign full taxonomic information to each identified taxon where possible. Unless otherwise stated, all analyses were performed using custom scripts in R.

### Phylogenetic tree

A phylogenetic tree of all sequences was generated using FastTree 2.1^28^, using the general time reversible (GTR) + CAT model and default parameters. The tree was rooted using the archaeal sequences as an outgroup. Phylogenetic clades were obtained using the ‘Ancestor’ function in the ‘phangorn’ R package^29^. A relative abundance matrix, with abundance based on how many times sequences belonging to a phylogenetic clade appeared in a sample, was calculated.

### Ordination

Bray–Curtis dissimilarity and Jaccard Index values were calculated among all samples (based on the relative abundance matrix) and used for non-metric multidimensional scaling (NMDS) via the ‘MASS’^30^ and ‘ecodist’ R packages^31^, checking to ensure convergence in all cases.

### Machine learning

Random forest (RF) models were run using the ‘randomForest’ package^32^ in R. Specifically, the clade relative abundance matrix was used as an input for the RF, using a forest of 100,000 trees and the mtry value was left at default settings (the square root of the number of clades). Separate forests were run to predict whether a sample was 6 or 18 weeks old, whether a sample was stool or mucus, whether it was a WT or an *mdr1a*^*−/−*^ sample, what cage the sample was taken from and the mother of each respective offspring. Each forest was controlled for all other treatments (i.e. a random forest predicting age included genotype and microbial niche as explanatory variables, in addition to the generated clades). The ‘MeanDecreaseAccuracy’ (MDA) value was used as a measure of how important each clade (or treatment) was at predicting treatment information and the out-of-bag (OOB) error rate was used to determine the predictive accuracy of the model. Nodes were ranked based on MDA value, taking the five most important nodes, determining the descendant tips and confirming the identity of the tip sequences via the BLAST+ results^26^. Additionally, the depth of each node was determined using the ‘distances’ function in the igraph R package^33^. A phylogenetic tree annotated with the resulting information was plotted using the ‘plot.phylo’ function in the ‘ape’ package^34^.

### Model validation

In order to validate each model, we included a ‘randomised’ negative control RF where relative abundances of each node were permuted with respect to each sample and the predictive accuracy was assessed. In addition, we took the relative abundances of an important node for age and redistributed the abundance to only WT samples. The RF was repeated to investigate whether this node would appear as important for genotype. We also ran RF’s with an increasing number of trees, using three different random seeds and performed Spearman’s Rank correlation on the MDA values obtained among each set of three RFs of the same size. The Monod/Michaelis–Menten model was fitted, to determine how an increasing number of trees affected correlation of the MDA values. Finally, we included technical replicates of one stool sample that was used as an internal control between sequencing runs, in our forest models. We examined the MDA values for all the clades in each of these replicates to see how tightly correlated they were. Additionally, we compared the predictive accuracy of the RF model when using these different replicates.

### Statistical analysis

Analysis of the real vs null RF models predictive accuracy was performed using 2-way ANOVA, with a Sidak’s post hoc test in GraphPad Prism 8 (GraphPad Software, La Jolla, USA). Permutational multivariate analysis of variance (PERMANOVA) was used to determine interactions between taxa and treatment groups, using the ‘adonis’ function in the vegan R package^35^. Effects of age, niche, genotype and all possible interactions were considered. All significant effects (P < 0.05) are reported.

## Results

### Phylogenetic tree of 16S rDNA data derived from the gut microbiota

Microbiota samples from the stools and colonic mucus (Fig. 1a) of 20 male mice were collected from two genotypes (WT vs colitis prone-*mdr1a*^*−/−*^), at two different time points (6 vs 18 weeks of age, with different mice sampled at each time point). By the later time point, *mdr1a*^*−/−*^ animals had developed a mild or moderate colitic phenotype (see methods). Littermates of the different genotypes were co-housed in 8, mixed genotype cages (Supplementary Figure S1). On average, 10,442 16S sequences (range 1892–25,681) were obtained per sample. All sequences were used to create a phylogenetic tree, comprising 496,550 tips and 428,234 internal nodes, which separated the major phyla (Fig. 1b). Sequences derived from stool and mucus were distributed across the tree (Supplementary Figure S2a), as were sequences associated with other criteria (age, genotype and cage) (Supplementary Figure S2b–d).

### Strong separation of the gut microbiota by microbial niche, age and cage but not host genotype

**Figure 2:**
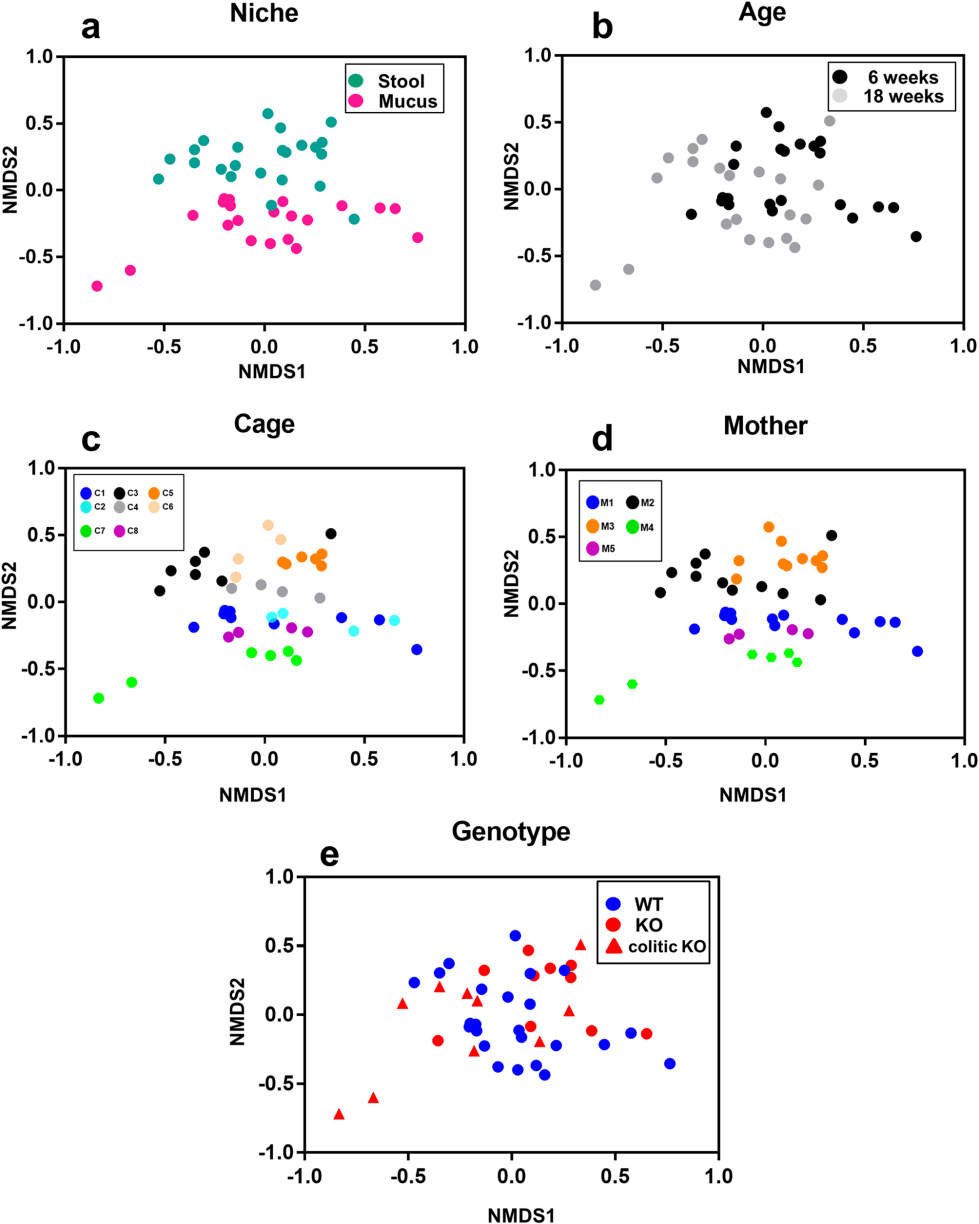
Separation of microbiota via NMDS for microbial niche, age, cage and mother. Two dimensional non-metric multidimensional scaling (NMDS) was performed using a Bray Curtis dissimilarity matrix based on the relative abundance of all clades in the phylogenetic tree shown in Fig. 1B. Plots highlighting stool and mucus samples (**a**), 6 and 18 week old samples (**b**), different cages (**c**, C1–C8 represent cages 1–8), mothers (**d**, M1–5 represent mothers 1–5) and WT (wildtype) and KO (*mdr1a*^*−/−*^) samples (**e**) are illustrated. Each point corresponds to a stool or a mucus sample. These samples were taken from n = 10 mice per genotype. Figure produced in GraphPad Prism 8.

To avoid bias by taxonomic level, we constructed a data matrix comprising the relative abundance [(number of tips in clade in sample)/(total number of tips in sample)] of clades corresponding to all 428,234 internal nodes of the phylogenetic tree in each of our samples. This avoided assigning OTUs, or using a reference database. To visualise the major differences in the microbial communities in an unsupervised fashion, Bray–Curtis dissimilarity values were calculated from our data matrix between all samples and used as an input for a 2-dimensional non-metric multidimensional scaling (NMDS) ordination (Fig. 2). A stress plot for this ordination (Supplementary Figure S3a), shows a stress value of 0.18, within the 0.20 acceptability threshold^36^. There was clear separation of samples by niche (PERMANOVA, p = 0.0001) (Fig. 2a). There was less visual separation by mouse age (6 vs 18 weeks, PERMANOVA, p = 0.0001) (Fig. 2b) which is in concordance with our previous work^17^. Samples from the same cage localised closely in the ordination. Cages containing different litters from the same mother (see Supplementary Figure S1) were adjacent or overlapping in the plot, suggesting maternal effects influencing, but not fully explaining, cage-specific microbiomes (Fig. 2c,d). Little separation was found when comparing genotypes or colitic phenotype with no significant effect on its own (PERMANOVA, p = 0.36) or in interaction with age (p = 0.07) or niche (p = 0.1) (Fig. 2e). A similar analysis using only the presence/absence of taxa (Jaccard Index) gives similar conclusions for niche (PERMANOVA, p = 0.01) and age (PERMANOVA, p = 0.001), but visually separates treatments less clearly (Supplementary Figure S4). As with the Bray Curtis matrix, genotype had no significant effect on its own (PERMANOVA, p = 0.38) or in interaction with age (p = 0.38) or niche (p = 0.97). The stress value was 0.15 (Supplementary Figure S3b).

### Specific microbiota are strongly associated with age, microbial niche, cage and mother but not host genotype

**Figure 3:**
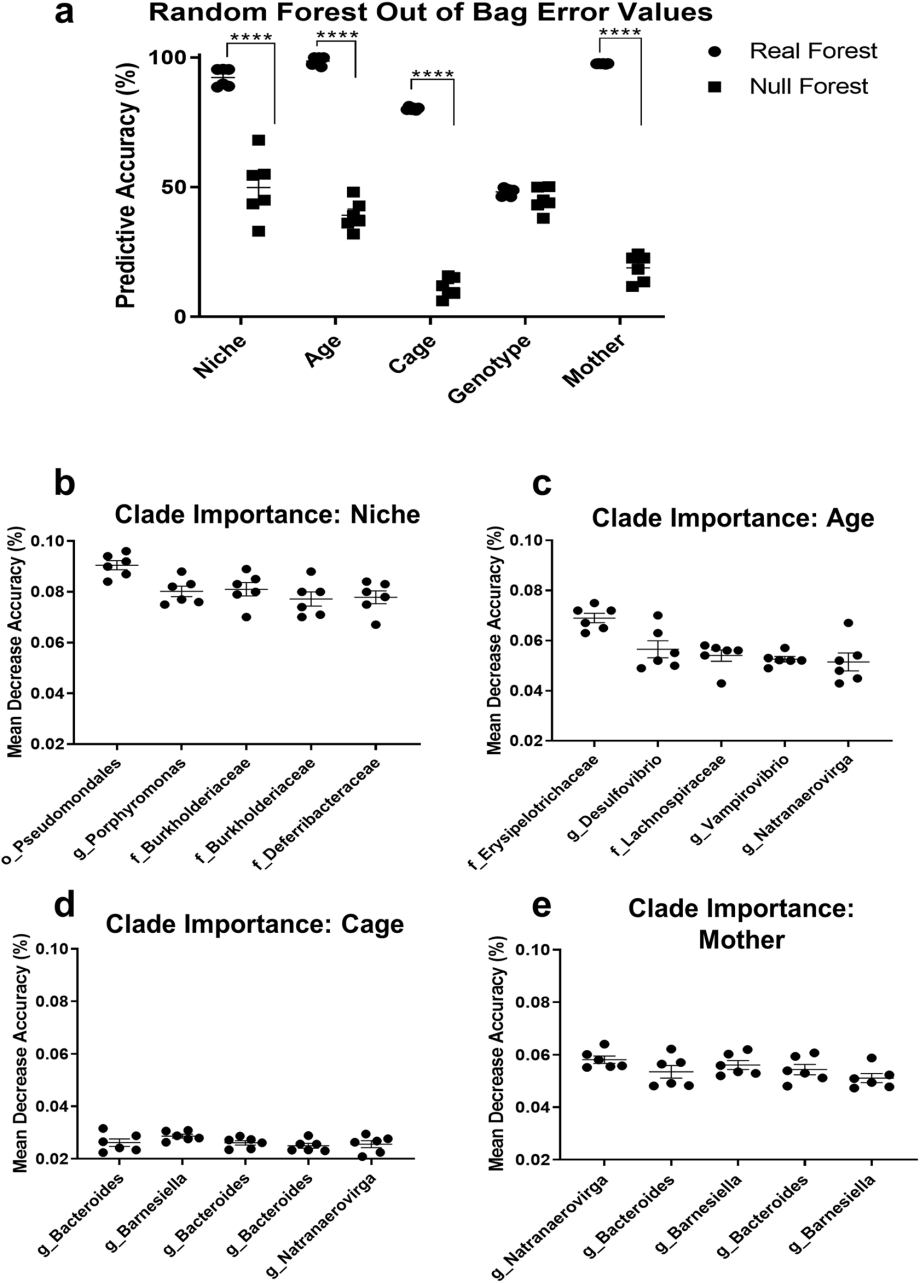
Random forest model identifies strong associations between the microbiota, niche, age and cage. The predictive accuracy of the random forest model at taking a sample and discriminating between the different treatment groups is shown (**a**). The five most important nodes associated with microbial niche (**b**), host age (**c**), social group (cage) (**d**) and mother (**e**), are named based on the finest scale taxon containing the closest BLAST hits of all sequences in the clade. Taxa are prefixed with their taxonomic level: order (o_), family (f_) and genus (g_). Bars represent means and standard errors. Asterisks represent significance determined using Two Way ANOVA-Sidak’s post hoc: *P* < 0.00001 (****). Similar plots for further validation control analyses are shown in Supplementary Figure S3 and S4. n = 6 technical replicates. Figure produced in GraphPad Prism 8.

To determine the taxa driving the observed differences in community structure, the relative abundance matrix was used to construct machine learning models (random forests, RFs). Separate RF models were created to identify age, genotype, niche, cage and mother based on the relative abundance of the clades (as defined by the phylogenetic tree, Fig. 1b) in each sample. These models were compared against a null (negative control) model where relative abundances were permuted among taxa within samples to remove true associations, while keeping the characteristics of the individual samples (e.g. those arising from any differences in coverage). Further testing to validate the reproducibility of this approach is in the Supplementary Information (comprising Supplementary Figures S5–S7). Niche was determined from the microbiota with 92% accuracy, age and mother with ~ 98% accuracy and cage with 80% accuracy (averaged across six technical replicates), in all instances substantially higher than the negative control model (Fig. 3a) (Two Way ANOVA-Sidak’s post hoc test: *P* < 0.0001). Genotype could not be determined from the microbiota using our RF models any better than in the negative control (Fig. 3a). Models considering genotype were therefore not considered further.

The RFs give an importance value for each clade (the tree’s internal nodes) in discriminating between groups. To identify which bacteria the clades encompassed, we used BLAST+ on all sequences, recording the taxonomic identity of the top hit (hits that had a percentage coverage < 100% were discarded). The finest-scale taxonomic grouping containing all sequences descending from the five most important clades is shown in Fig. 3b–e for niche, age, cage and mother RFs respectively. The named clades do not represent all bacteria within that taxon, rather they represent specific bacteria, all of which fall within the taxon. For microbial niche, the most important distinguishing clades were all Gram negative and mostly comprised *Proteobacteria*: the order *Pseudomonadales* and three clades in the families *Burkholderiaceae* and *Deferribacteraceae* and all these clades were more abundant in the mucus samples (Supplementary Figure S8a). The *Deferribacteraceae* containing clade mostly comprised *Mucispirillum*, a known mucus-associated bacteria^37^. The genus *Porphyromonas* was more abundant in stool samples. The most important clades separating ages were the families *Erysipelotrichaceae* and *Lachnospiraceae* within the *Firmicutes* phylum (which have each been specifically associated with young mouse microbiomes before^38^) plus three genera: *Natranaerovirga, Desulfovibrio*, and *Vampirovibrio* in the *Firmicutes*, *Proteobacteria* and *Cyanobacteria* phyla respectively*.* With the exception of *Natranaerovirga*, all these bacteria were prevalent in the 18 week old mice (Supplementary Figure S8b). The most important clades separating cages and mothers were *Natranaerovirga* (a different clade from that separating ages) plus four clades within the order *Bacteroidales*—three comprising the genera *Bacteroides* and one comprising *Barnesiella* (Supplementary Figure S8c,d).

### Abundant, low-level taxa distinguish cage and maternal microbiomes but not age or niche

**Figure 4:**
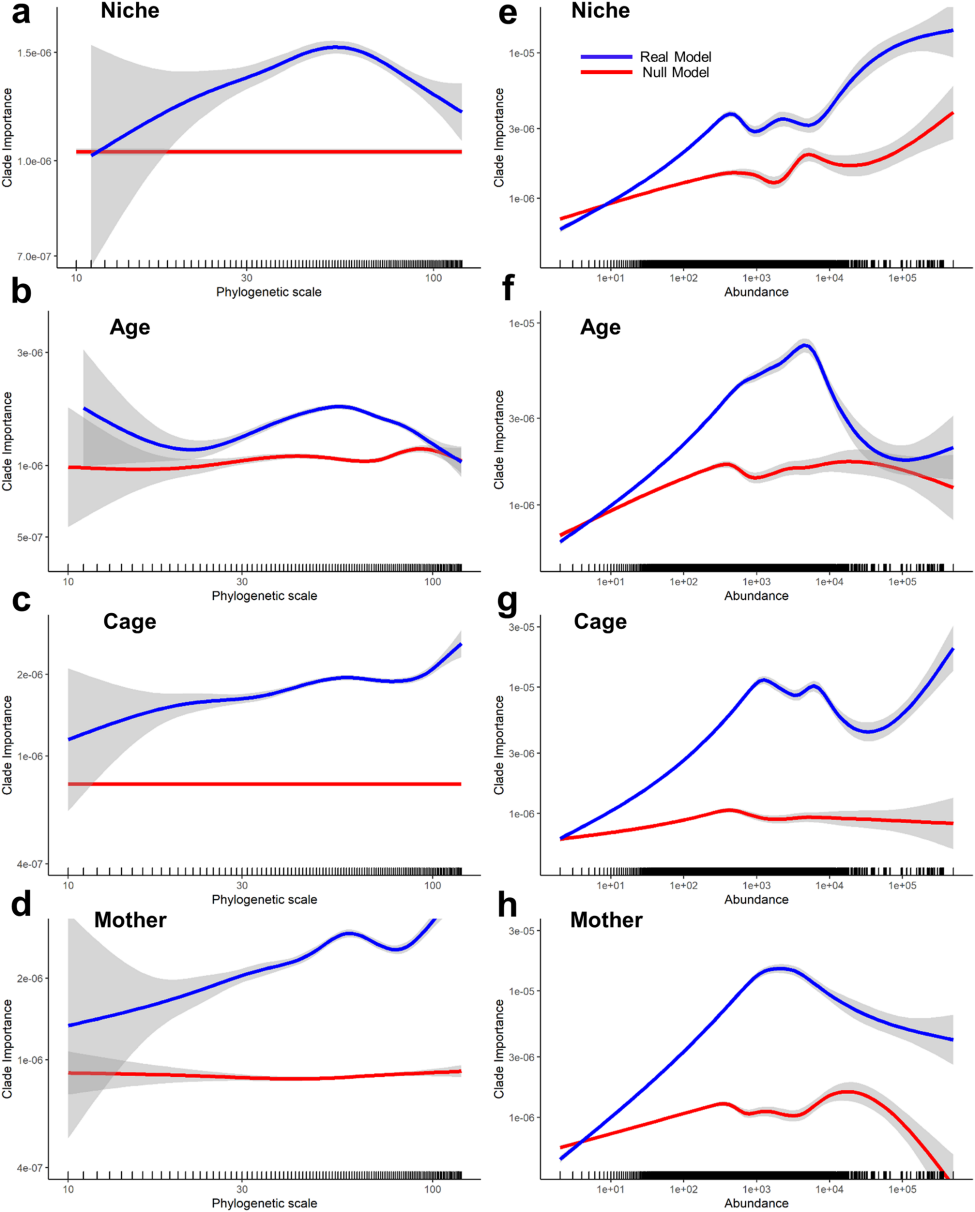
Abundant, low-level taxa distinguish cage microbiomes but not age or niche. The phylogenetic scale of each clade was measured as the number of nodes in the phylogenetic tree between the clade and root (small values associated with large-scale taxa such as phyla). Phylogenetic scale was compared against the ‘mean decrease in accuracy’ (MDA) value when running a random forest that distinguished the niche (**a**), age (**b**), cage (**c**) and mother (**d**). Clade abundance was measured as the number of sequences (tips) descending from each clade. Each clade’s abundance was compared against its MDA value, when running a forest that distinguished the niche (**d**), age (**e**), cage (**f**) and mother (**g**). The smoothed mean for the ‘real’ random forest model is illustrated in blue and for a null (negative control) random forest model in red. The grey areas refer to confidence intervals. Each small vertical ‘rug’ line above the horizontal axis indicates the location of a single taxon. Note the logarithmic scales on all axes. Similar plots using a different measure of phylogenetic scale (distance of clades from the root of the tree) are in Supplementary Figure S5. Figure produced in R 3.6.0 for Windows.

Having identified taxa at different phylogenetic levels as particularly important for separating microbiomes, we looked systematically at the phylogenetic scales that are important for separating different microbiomes. Clade importance was analysed as a function of the number of nodes between the clade and the root of the phylogenetic tree (Fig. 4) or the distance from each clade to the root (Supplementary Figure S9). These measures distinguish clades close to the root (high-level taxa with fewer nodes and shorter branch length from the base of the clade to the root) corresponding, e.g. to phyla, and clades far from the root (low-level taxa with more nodes and longer branch lengths from the base of the clade to the root) corresponding e.g. to genera. For both age and niche, neither the lowest nor the highest level clades were consistently important but the most clearly important clades were of intermediate taxonomic levels (Fig. 4a,b). For age, this separation between true and null models differs between the metrics: while the intermediate level taxa are important across both metrics, the highest level clades are the most important with the distance metric (Supplementary Figure S9b vs. Fig. 4b). However, for differences among cages and mothers, while intermediate level clades were important, many of the most important groups were at the extreme of low level taxa, i.e. differences in sub-specific groupings (Fig. 4c,d, Supplementary Figure S9c,d).

The number of sequences within a clade of the tree that were present in a particular set of samples is an estimate of its abundance in that microbiome. We therefore asked how abundance of bacterial taxa correlated with its importance in distinguishing microbiomes. We found that, for separating niche, age, cage or maternal microbiomes, moderately abundant taxa were important, whereas the rarest taxa were never important (Fig. 4e–h). The most abundant taxa were important for distinguishing cage and maternal microbiomes (Fig. 4g,h) but much less so for distinguishing different ages or niches (Fig. 4e,f) where the true and null models converge on the right).

Finally, we asked how the importance of particular taxa related across models distinguishing the different criteria. The distributions of importance values are wide for all RFs, including null models (Supplementary Figure S10). Nonetheless, we expect a positive association between the importance of particular taxa in distinguishing cages and in distinguishing mothers, since individual litters were housed in separate cages (Fig. 2). These are indeed associated (rank correlation of 0.291, cf. 0.05 for the null model, Supplementary Figure S10). However, the importance of particular taxa was uncorrelated across all other forests that distinguished different criteria (age versus niche, age versus cage, age vs mother, niche versus cage and niche vs mother had rank correlations − 0.002, 0.005, 0.002, − 0.001 and − 0.0007 respectively, each less than their respective null models). Thus, while we can identify broad trends (Fig. 4) and a few key taxa associated with particular gut features (Fig. 3), we did not find widely applicable ‘indicator’ taxa that were individually sensitive to multiple effects on the gut microbiome.

## Discussion

We were able to discriminate clearly between the microbiomes of 6 and 18 week old mice, mucus and stool samples, different groups of mixed genotype co-housed mice and mice with different mothers. This confirms the robustness of our models as it is consistent with others’ work, for instance showing microbiome changes with age in both humans and mice^39,40^, work identifying microbial niche as the strongest factor for separation of the microbiota^17,41^ and the impact of environment on the microbiome^4,11,42^. We associated the microbiota with cage with approximately 80% accuracy, suggesting that each cage has a distinct microbial signature. Mice were housed according to their litter and the mother could be distinguished with 98% accuracy, so the maternal microbiome is likely to be critical in determining this signature. Differences in environment can impact the microbiome and have functional impacts on the host, including altered permeability of the mucus barrier^4^. However, the risk of confounding phenotypes of interest with environmental variation is much broader—in humans, the microbiota is also both vertically transmissible (from mother to offspring) and horizontally transmissible (between household members)^43^. Nonetheless, it is predominantly animal studies that are used to assign causality of changes in the microbiome to phenotypic effects. Therefore, it is imperative that environmental factors, including both mothers and housing, are both reported and controlled for. Currently, this is by no means universal^12^.

Our mice are still relatively young, with initial samples taken only ~ 3 weeks after weaning and at 18 weeks old. Therefore the strong differences seen by age may be due to the microbiota still adjusting at 6 weeks due to changed diet from milk to solid food. Solid food may itself contain plant and bacterial sequences (including plant sequences that may be mistaken by taxonomy databases for cyanobacteria^44^). It is also true that, even in our experimental design, because it is only possible to obtain a single mucus sample from a mouse, we have confounded the age effect with the cage and maternal effects, meaning that we cannot exclude the possibility that the true effect of age is in fact less distinct. Nonetheless, dietary-derived microbial changes in mice can happen within a much shorter timeframe^45,46^, and the nature of the age effect identified (Figs. 3c and 4b,f) is clearly distinct from the cage and maternal effects, consistent with it being a distinct age effect. The fact that we saw these differences clearly validates our approach to modelling them. In addition, the microbes identified as key to these changes include expected taxa such as the family *Erysipelotrichaceae*. This family distinguished our older mice (Fig. 3c) and has been associated with the development of IBD (both positively and negatively)^47,48^ and colorectal cancer^49^.

We found no consistent differences between the gut microbiomes of wildtype and colitis-prone (*mdr1a*^*−/−*^) genotypes co-housed together in mixed genotype cages. Differences in the microbiota of WT and *mdr1a*^*−/−*^ mice *have* been reported^17,50^ (with and without littermate controls respectively) so the fact that we do not see them here (Figs. 2e, 3a) is unexpected. Discrepancies in sample size between treatment groups can be a problem for RFs applied to such data^51^ and machine learning typically uses much larger sample sizes. However, here sample sizes are well balanced (10 wildtype and 10 *mdr1a*^*−/−*^ mice with 2 samples from each, albeit individual mothers gave rise to different numbers of offspring, Supplementary Figure S1) and sufficient for distinguishing other criteria. The older *mdr1a*^*−/−*^ mice were starting to develop colitis. Therefore, changes in the microbiome with colitis may have obscured any consistent differences among genotypes. Alternatively, previous analyses may have been misled by large cage effects (Figs. 2c, 3a) into erroneously attributing some of that variation to differences among genotypes. For instance, studies finding differences between the stool microbiota of eosinophil-deficient mice compared to wildtype mice (e.g.^52^) do not report controls for cage effects, e.g. via littermate controls. Thus, they cannot rule out the possibility that differences in the microbiota are due to environmental effects.

Changes in the gut microbiome, at any taxonomic level have been attributed as leading to functional impacts on the host. For instance, a reduction in the abundance and diversity of *Firmicutes* is associated with IBD in human patients^53–55^ and *Bacteroidetes* has been shown to be both increased^56^ and decreased^53^ with respect to inflammation. However, our data did not find such high-level taxa to show consistent differences in any of our microbiome comparisons (Fig. 4a–d, Supplementary Figure S9). This could be because our phylogenetic tree does not fully capture the relationships among the highest level taxa (Fig. 1b), because there is limited phylogenetic information in amplicons from the subset of the 16S rRNA used. Even trees using the complete 16S rRNA sequence from carefully chosen bacteria do not fully capture their evolutionary history^57^ and partial 16S rRNA trees can only be made to agree with accepted evolutionary relationships by incorporating many constraints (as done by Louca et al.^58^). Here, we did not want to create the biases that such constraints would impose. Even taking this approach, phyla themselves are largely resolved (Fig. 1b), so inadequacies in the tree seem unlikely to account for the lack of consistent differences in these taxa among criteria (Fig. 4a–d, Supplementary Figure S9). These high-level taxa are also abundant taxa. While there has been a focus on the importance of rare taxa (e.g.^59^), a priori, it might have been reasonable to expect that the more abundant taxa would have the most important functional consequences for the host (as posited in other microbiomes e.g.^60^) and therefore be the most likely to differ between different circumstances. However, the only microbiome comparison in which we find the most abundant taxa to be important was in distinguishing among cages and mothers. In those cases, it was low-level taxonomic groupings (e.g. clades within the abundant genus *Bacteroides*), not phyla, that distinguished cage-specific microbiomes. This findings suggests the importance of relatively rare microbial species.

Rare bacterial species are thought to play a large role in a range of ecosystems, including host and environmental microbiomes^61,62^. Specifically, rare taxa have been associated with inflammation^63^. Here however, we did not find the rarest taxa to be important in discriminating between microbiomes (Fig. 4e–h). This could be an artefact of the fact that, almost by definition in a complex microbiome, rare taxa are likely to be missed from at least a subset of samples through random sampling. Therefore, rare taxa would not show consistent differences among the factors considered (niche, age, cage or mother), as we find (Fig. 4e–h).

The presence or absence of certain taxa will allow other bacterial families/species to flourish or be inhibited, which in turn will alter host/microbial homeostasis, emphasising the need to consider communities and not bacteria in isolation. Furthermore, changes in one bacterium may not be significant functionally if the clade as a whole is unaffected. RF models can account for such interactions among taxa, and the ‘importance’ assigned to a taxon (Fig. 3b–e) takes these into account^64^. RF approaches have previously proved effective where ratios of Firmicutes to Bacteroidetes could not^51^ and could discriminate between patients with active Crohn’s disease and those in remission with ~ 70% accuracy^65^. Here we go one step further, by using the full range of clades in a phylogenetic tree as explanatory variables in the RF model. This avoids over-stretching the data by assigning a sequence read to one taxon rather than another, when it is in fact similarly close or distant to both. It also ensures that we do not lose power that is in the data e.g. clear phylogenetic structure among sequences that are closer than a given threshold (typically 97% identity used for OTUs^66^). The development of ‘de-noising’ approaches such as DADA2^67^ and DEBLUR^68^ to generate amplicon sequence variants (ASVs) also goes some way to avoiding the problems of using universal similarity thresholds to define OTUs. However, different de-noisers can lead to different results^69^. We avoid such issues by using all sequence variants, whether true ASVs or sequencing errors. Given our well-controlled experiment, we do not expect different sequencing errors in different treatments. This expectation is consistent with the fact that we find the very rarest variants, which will be highly enriched for sequencing errors, are no better than random at distinguishing any of our treatments (Fig. 4e–h). This approach and our focus on differences among treatments comes at a cost—we do not even attempt to estimate the ‘true’ community composition of any particular sample or how well it’s captured by the data. Despite this, we are able to identify clear compositional differences and phylogenetic patterns in communities across the treatments studied.

In conclusion, taking a carefully designed factorial experiment involving co-housing of different genotypes of littermate mice, we have been able to identify major changes in the gut microbiome with age, niches, cages and mothers, but not genotype (Figs. 2, 3a). In particular, we highlight a clear impact on the gut microbial communities associated with these experimental factors, particularly cage and maternal effect with phylogenetic patterns that are in stark contrast to niche and age. Together, this work reveals the subtlety of the balance between homeostasis and difference in the gut microbiome, and emphasises the need to carefully account for host environment when performing future studies.

## Supplementary Information

Supplementary Figures.

Supplementary Information.

## Data Availability

The sequence data analysed during the current study is available on the European Bioinformatics Institute (EBI, https://www.ebi.ac.uk/ena) (study accession number PRJEB6905). Code to reproduce the main text figures produced in R will be available on FigShare (10.48420/13649837).

## References

[1] Lynch JB, Hsiao EY (2019). Microbiomes as sources of emergent host phenotypes. Science (New York, N.Y.).

[2] Hiergeist A, Reischl U, Gessner A (2016). Multicenter quality assessment of 16S ribosomal DNA-sequencing for microbiome analyses reveals high inter-center variability. Int. J. Med. Microbiol..

[3] Li H (2015). The outer mucus layer hosts a distinct intestinal microbial niche. Nat. Commun..

[4] Jakobsson HE (2015). The composition of the gut microbiota shapes the colon mucus barrier. EMBO Rep..

[5] Papa E (2012). Non-invasive mapping of the gastrointestinal microbiota identifies children with inflammatory bowel disease. PLoS ONE.

[6] Kalliomaki M, Collado MC, Salminen S, Isolauri E (2008). Early differences in fecal microbiota composition in children may predict overweight. Am. J. Clin. Nutr..

[7] Singh G, Brass A, Knight CG, Cruickshank SM (2019). Gut eosinophils and their impact on the mucus-resident microbiota. Immunology.

[8] Hoy YE (2015). Variation in taxonomic composition of the fecal microbiota in an inbred mouse strain across individuals and time. PLoS ONE.

[9] Song SJ (2013). Cohabiting family members share microbiota with one another and with their dogs. Elife.

[10] Stappenbeck TS, Virgin HW (2016). Accounting for reciprocal host–microbiome interactions in experimental science. Nature.

[11] Hildebrand F (2013). Inflammation-associated enterotypes, host genotype, cage and inter-individual effects drive gut microbiota variation in common laboratory mice. Genome Biol..

[12] Bramhall M, Florez-Vargas O, Stevens R, Brass A, Cruickshank S (2015). Quality of methods reporting in animal models of colitis. Inflamm. Bowel Dis..

[13] Choo JM (2017). Inbred mouse populations exhibit intergenerational changes in intestinal microbiota composition and function following introduction to a facility. Front. Microbiol..

[14] Ley RE (2005). Obesity alters gut microbial ecology. Proc. Natl. Acad. Sci. U.S.A..

[15] Million M (2012). Obesity-associated gut microbiota is enriched in *Lactobacillus**reuteri* and depleted in *Bifidobacterium**animalis* and *Methanobrevibacter**smithii*. Int. J. Obes..

[16] Stevens JR, Jones TR, Lefevre M, Ganesan B, Weimer BC (2017). SigTree: A microbial community analysis tool to identify and visualize significantly responsive branches in a phylogenetic tree. Comput. Struct. Biotechnol. J..

[17] Glymenaki M (2017). Compositional changes in the gut mucus microbiota precede the onset of colitis-induced inflammation. Inflamm. Bowel Dis..

[18] Schinkel AH (1994). Disruption of the mouse mdr1a P-glycoprotein gene leads to a deficiency in the blood-brain barrier and to increased sensitivity to drugs. Cell.

[19] Schneider CA, Rasband WS, Eliceiri KW (2012). NIH Image to ImageJ: 25 years of image analysis. Nat. Methods.

[20] St John, J. *SeqPrep*. https://github.com/jstjohn/SeqPrep (2018).

[21] Meyer F (2008). The metagenomics RAST server—A public resource for the automatic phylogenetic and functional analysis of metagenomes. BMC Bioinform..

[22] Cox MP, Peterson DA, Biggs PJ (2010). SolexaQA: At-a-glance quality assessment of Illumina second-generation sequencing data. BMC Bioinform..

[23] Nawrocki EP, Eddy SR (2013). Infernal 1.1: 100-fold faster RNA homology searches. Bioinformatics.

[24] Cole JR (2014). Ribosomal Database Project: Data and tools for high throughput rRNA analysis. Nucleic Acids Res..

[25] R Core Team. *R: A Language and Environment for Statistical Computing*. https://www.r-project.org/ (2016).

[26] Camacho C (2009). BLAST+: Architecture and applications. BMC Bioinform..

[27] Chamberlain SA, Szocs E (2013). taxize: Taxonomic search and retrieval in R. F1000Research.

[28] Price MN, Dehal PS, Arkin AP (2010). FastTree 2—Approximately maximum-likelihood trees for large alignments. PLoS ONE.

[29] Schliep KP (2011). phangorn: Phylogenetic analysis in R. Bioinformatics.

[30] Venables, W. & Ripley, N. *Modern Applied Statistics with S.* 4th edn (2002).

[31] Goslee, S. C. & Urban, D. L. The ecodist package for dissimilarity-based analysis of ecological data. **22**, 1–19. https://www.jstatsoft.org/index.php/jss/article/view/v022i07 (2007).

[32] Liaw A, Wiener M (2002). Classification and Regression by randomForest. R News.

[33] Csard, G. & Nepusz, T. *The igraph software package for complex network research*. http://igraph.org (2006).

[34] Paradis E, Claude J, Strimmer K (2004). APE: analyses of phylogenetics and evolution in R language. Bioinformatics.

[35] Oksanen, J. *et al. vegan: Community Ecology Package*. https://CRAN.R-project.org/package=vegan (2016).

[36] Clarke KR (1993). Nonparametric multivariate analyses of changes in community structure. Aust. J. Ecol..

[37] Rodriguez-Pineiro AM, Johansson ME (2015). The colonic mucus protection depends on the microbiota. Gut Microbes.

[38] Kim YG (2017). Neonatal acquisition of Clostridia species protects against colonization by bacterial pathogens. Science.

[39] Langille MG (2014). Microbial shifts in the aging mouse gut. Microbiome.

[40] Odamaki T (2016). Age-related changes in gut microbiota composition from newborn to centenarian: A cross-sectional study. BMC Microbiol..

[41] Eckburg PB (2005). Diversity of the human intestinal microbial flora. Science.

[42] McCafferty J (2013). Stochastic changes over time and not founder effects drive cage effects in microbial community assembly in a mouse model. ISME J..

[43] Korpela K (2018). Selective maternal seeding and environment shape the human gut microbiome. Genome Res..

[44] Di Rienzi SC (2013). The human gut and groundwater harbor non-photosynthetic bacteria belonging to a new candidate phylum sibling to Cyanobacteria. Elife.

[45] Kozik, A. J. Sex, age, and TNF influence the gut microbiota in a mouse model of TNBS colitis. *FASEB J.* **31**, 657.12. 10.1096/fasebj.31.1_supplement.657.12 (2017).

[46] Kearney SM, Gibbons SM, Erdman SE, Alm EJ (2018). Orthogonal dietary niche enables reversible engraftment of a gut bacterial commensal. Cell Rep..

[47] Craven M (2012). Inflammation drives dysbiosis and bacterial invasion in murine models of ileal Crohn's disease. PLoS ONE.

[48] Schaubeck M (2016). Dysbiotic gut microbiota causes transmissible Crohn's disease-like ileitis independent of failure in antimicrobial defence. Gut.

[49] Chen W, Liu F, Ling Z, Tong X, Xiang C (2012). Human intestinal lumen and mucosa-associated microbiota in patients with colorectal cancer. PLoS ONE.

[50] Nones K (2009). Multidrug resistance gene deficient (mdr1a^−^^/^^−^) mice have an altered caecal microbiota that precedes the onset of intestinal inflammation. J. Appl. Microbiol..

[51] Walters WA, Xu Z, Knight R (2014). Meta-analyses of human gut microbes associated with obesity and IBD. FEBS Lett..

[52] Chu VT (2014). Eosinophils promote generation and maintenance of immunoglobulin-A-expressing plasma cells and contribute to gut immune homeostasis. Immunity.

[53] Frank DN (2007). Molecular-phylogenetic characterization of microbial community imbalances in human inflammatory bowel diseases. Proc. Natl. Acad. Sci. U.S.A..

[54] Ott SJ (2004). Reduction in diversity of the colonic mucosa associated bacterial microflora in patients with active inflammatory bowel disease. Gut.

[55] Manichanh C (2006). Reduced diversity of faecal microbiota in Crohn's disease revealed by a metagenomic approach. Gut.

[56] Walker AW (2011). High-throughput clone library analysis of the mucosa-associated microbiota reveals dysbiosis and differences between inflamed and non-inflamed regions of the intestine in inflammatory bowel disease. BMC Microbiol..

[57] Vetrovsky T, Baldrian P (2013). The variability of the 16S rRNA gene in bacterial genomes and its consequences for bacterial community analyses. PLoS ONE.

[58] Louca S (2018). Bacterial diversification through geological time. Nat. Ecol. Evol..

[59] Benjamino J, Lincoln S, Srivastava R, Graf J (2018). Low-abundant bacteria drive compositional changes in the gut microbiota after dietary alteration. Microbiome.

[60] Delgado-Baquerizo M (2018). A global atlas of the dominant bacteria found in soil. Science.

[61] Shade A (2014). Conditionally rare taxa disproportionately contribute to temporal changes in microbial diversity. MBio.

[62] Jousset A (2017). Where less may be more: How the rare biosphere pulls ecosystems strings. ISME J..

[63] Powell N (2012). The transcription factor T-bet regulates intestinal inflammation mediated by interleukin-7 receptor+ innate lymphoid cells. Immunity.

[64] Touw WG (2013). Data mining in the Life Sciences with Random Forest: A walk in the park or lost in the jungle?. Brief. Bioinform..

[65] Tedjo DI (2016). The fecal microbiota as a biomarker for disease activity in Crohn's disease. Sci. Rep..

[66] Konstantinidis KT, Tiedje JM (2005). Genomic insights that advance the species definition for prokaryotes. Proc. Natl. Acad. Sci. U.S.A..

[67] Callahan BJ (2016). DADA2: High-resolution sample inference from Illumina amplicon data. Nat. Methods.

[68] Amir A (2017). Deblur rapidly resolves single-nucleotide community sequence patterns. mSystems.

[69] Nearing JT, Douglas GM, Comeau AM, Langille MGI (2018). Denoising the Denoisers: An independent evaluation of microbiome sequence error-correction approaches. PeerJ.

