# Supplementary material for: Cage and maternal effects on the bacterial communities of the murine gut
Source: Sci Rep. 2021 May 10;11:9841. doi: 10.1038/s41598-021-89185-5 (PMC8110963; doi:10.1038/s41598-021-89185-5)
Supplement: Supplementary file 1 — Supplementary Figures. [file 41598_2021_89185_MOESM1_ESM.pdf]

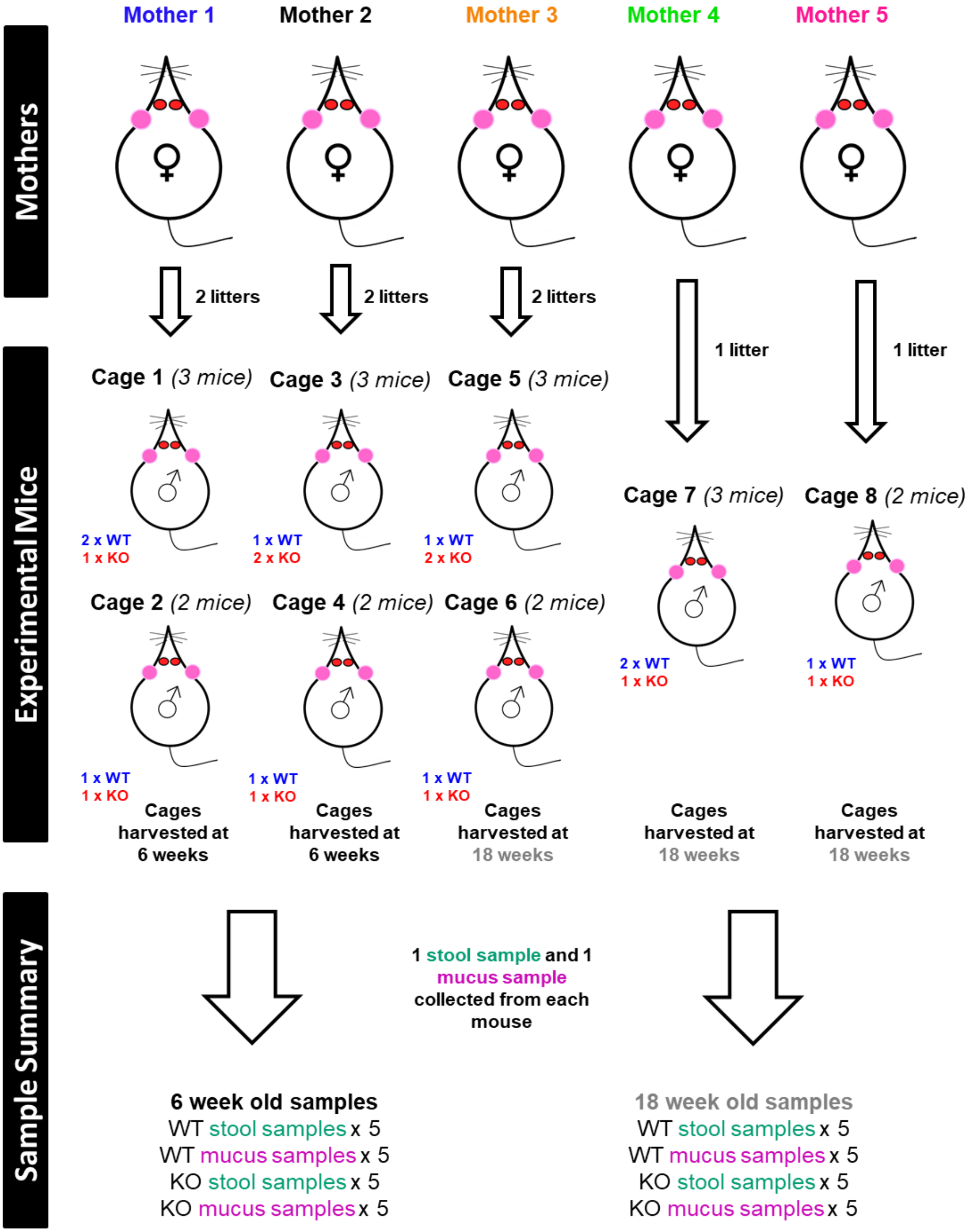

Supplementary Figure S1: Summary of experimental set up. In this study, there were 5 heterozygous mothers (FVB background), who had a total of 8 mixed-genotype litters, that gave rise to 20 experimental mice in total. Mice from each respective litter were co-housed and there was a mix of genotypes, wildtype (WT) and *mdr1a* knockout (KO) mice, in every cage. A stool and a mucus sample was collected from each mouse at either 6 or 18 weeks of age. Additionally, 4 technical replicate samples (3 from a cage 1 WT stool sample and 1 from a cage 5 WT mucus sample) were sequenced as internal controls.

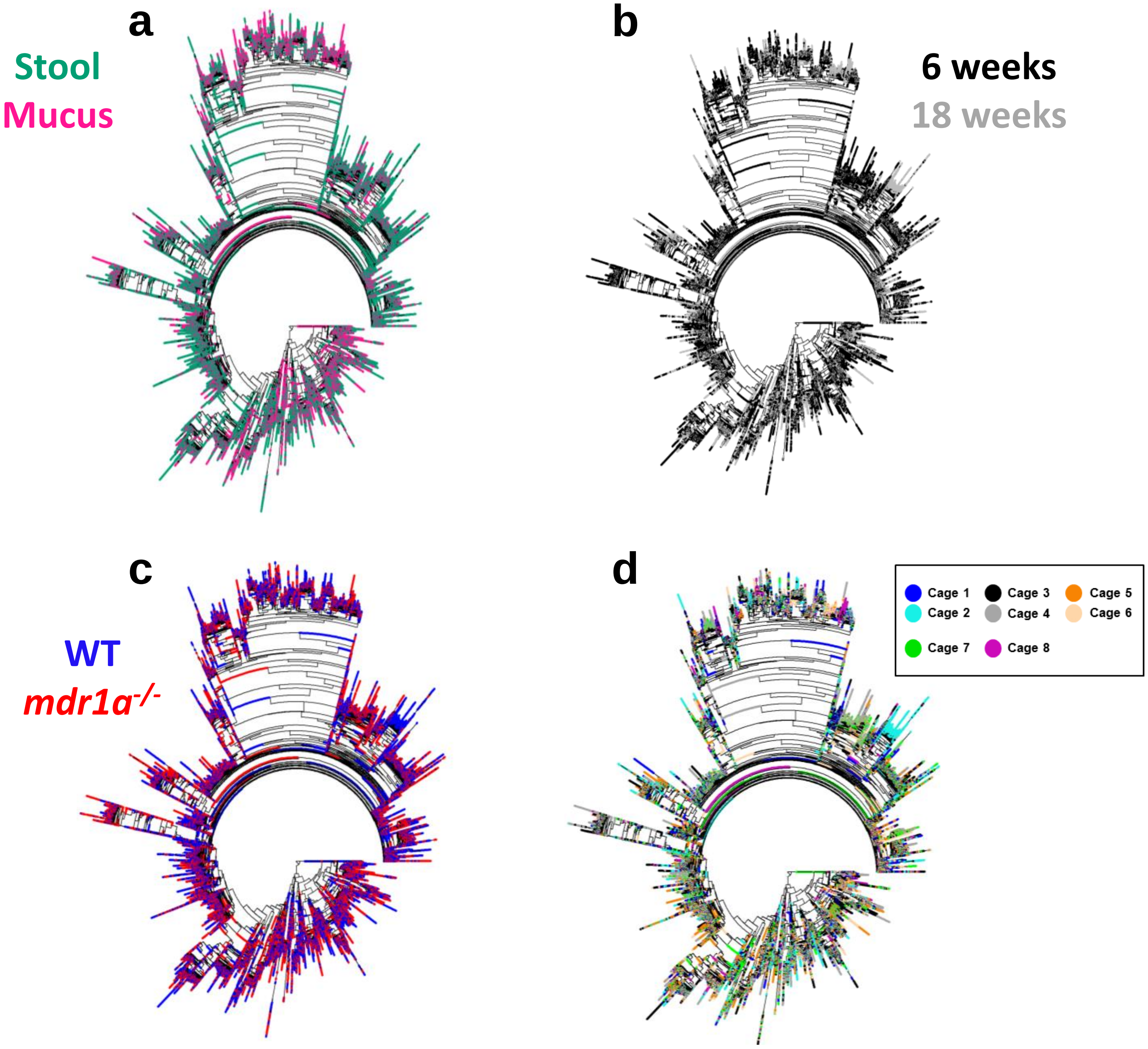

Supplementary Figure S2: Wide distribution of sequences on a phylogenetic tree when coloured by treatment groups. A phylogenetic tree of 16S rRNA sequences derived from the gut microbiota of FVB wildtype (WT) mice and *mdr1a*$^{-/-}$ mice was plotted and coloured by: Niche (a), age (b), genotype (c) and cage (d). Colours indicate stool (green), mucus (pink), 6 weeks of age (black), 18 weeks of age (grey), WT mice (blue), *mdr1a*$^{-/-}$ mice (red) and different cages (red, green, goldenrod, purple, dark blue, steel blue, pink and dark green). Figure produced in R 3.6.0 for Windows.

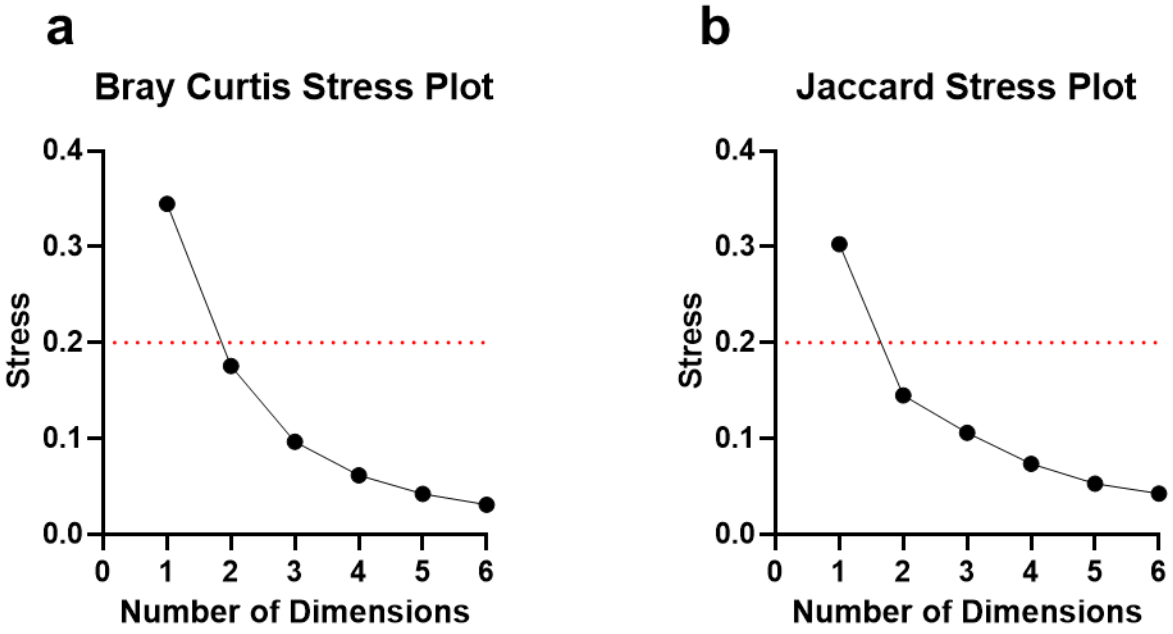

Supplementary Figure S3: Stress plots for NMDS. Two dimensional non-metric multidimensional scaling (NMDS) was performed using either a Bray Curtis dissimilarity matrix (Figure 2) (based on the relative abundance of all clades in the phylogenetic tree shown in Figure 1B) or Jaccard Index (based on the presence/absence of clades). Results of stress plots using Bray Curtis (a) or Jaccard Index (b), are shown. The 0.2 acceptability threshold is highlighted in red. Figure produced in GraphPad Prism 8.

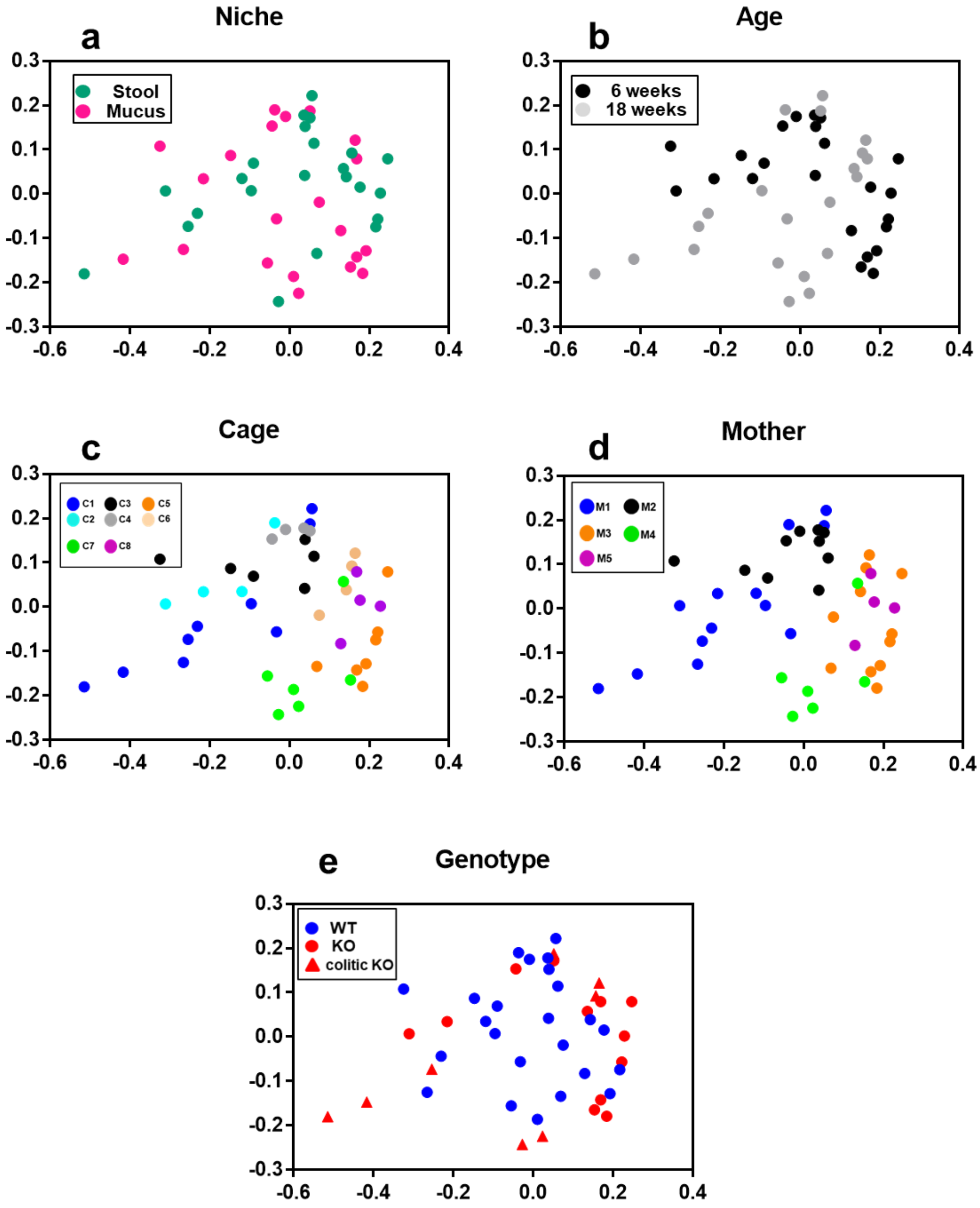

Supplementary Figure S4: NMDS derived from Jaccard Index. Two dimensional non-metric multidimensional scaling (NMDS) was performed using the Jaccard Index, based on the presence/absence of all clades in the phylogenetic tree shown in Figure 1B. Plots highlighting stool and mucus samples (a), 6 and 18 week old samples (b), different cages (c, C1-C8 represent cages 1-8), mothers (d, M1-5 represent mothers 1-5) and WT (wildtype) and KO (mdr1a-/-) samples (e) are illustrated. Each point corresponds to a stool or a mucus sample. These samples were taken from n = 10 mice per genotype. Figure produced in GraphPad Prism 8.

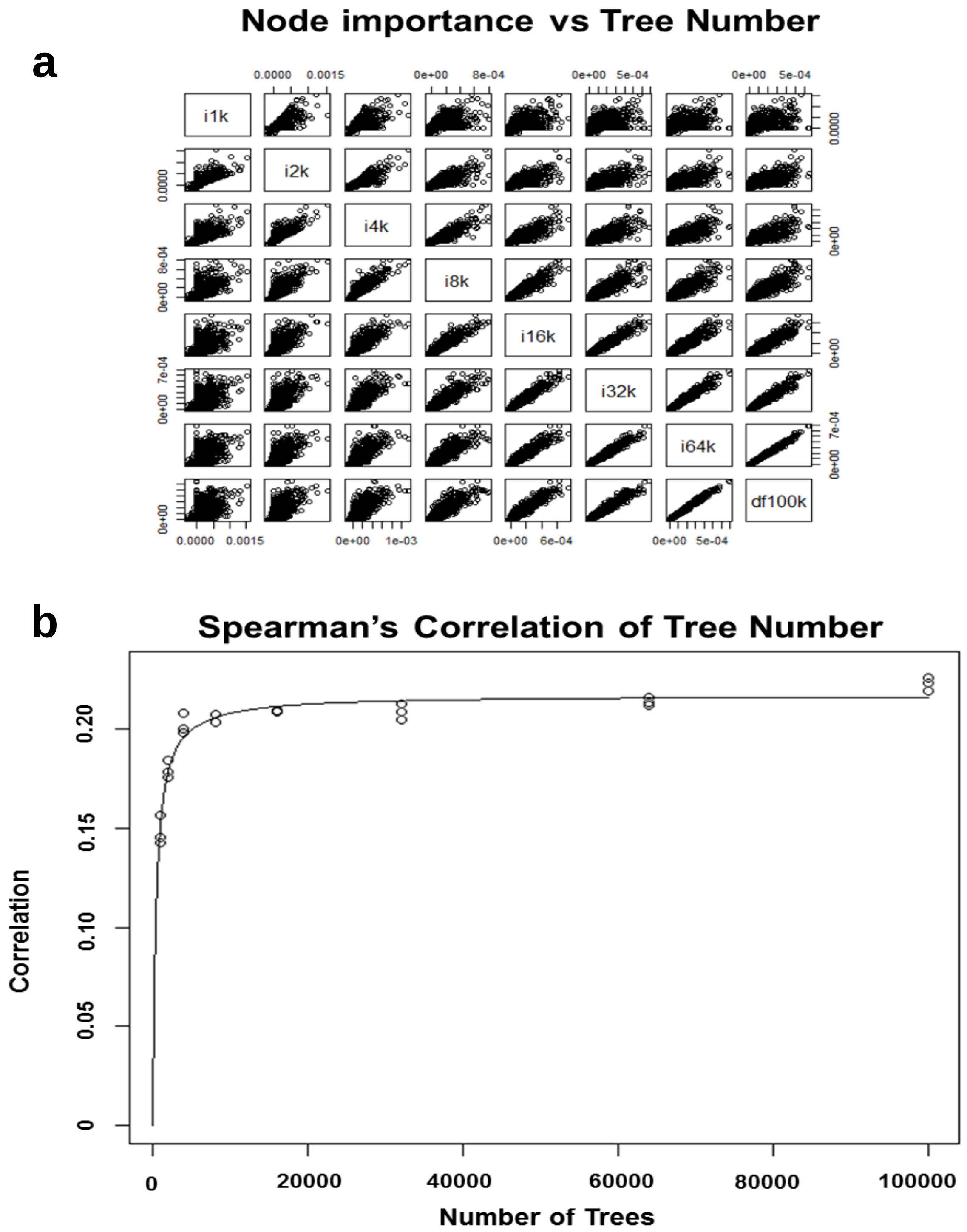

Supplementary Figure S5: Assessing Robustness of the RF model. A RF for separating stool and mucus was run using a particular random seed with an increasing number of trees in R and the 'MeanDecreaseAccuracy (MDA) value was plotted for each clade in all forests (a). Spearman's rank correlation between clade importance in three RFs separating stool and mucus, run with different random seeds and a particular number of trees. The best-fitting saturating (a Monod/Michaelis-Menten) curve is also shown (*Max* = 0.21, *K* = 439.97) (b). Figure produced in R 3.6.0 for Windows.

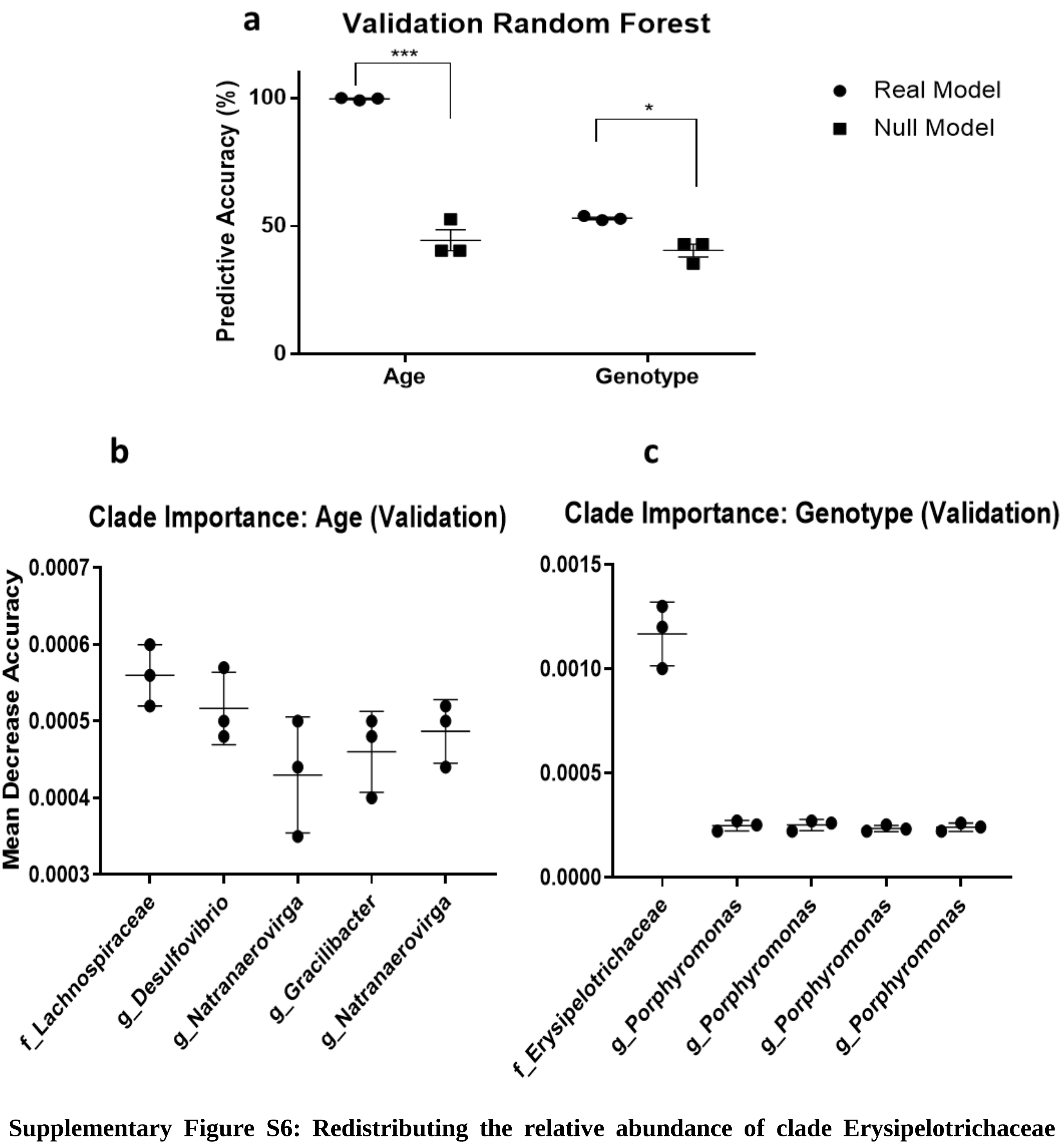

Supplementary Figure S6: Redistributing the relative abundance of clade Erysipelotrichaceae confers importance to genotype. The most important clade for age was identified and its relative abundances were redistributed into WT samples only and the RF was repeated. The predictive accuracy of the RF model at taking a sample and discriminating between age and genotype are displayed (a). The five most important nodes when comparing age (b) and genotype (c) via the RF are illustrated. Taxa are prefixed with their taxonomic level: family (f_) and genus (g_) Data shown as mean +/- standard error mean (SEM). Asterisks represent significance determined using Two Way ANOVA: $p = < 0.05$ (*) and $p = < 0.0001$ (***). Figure produced in GraphPad Prism 8.

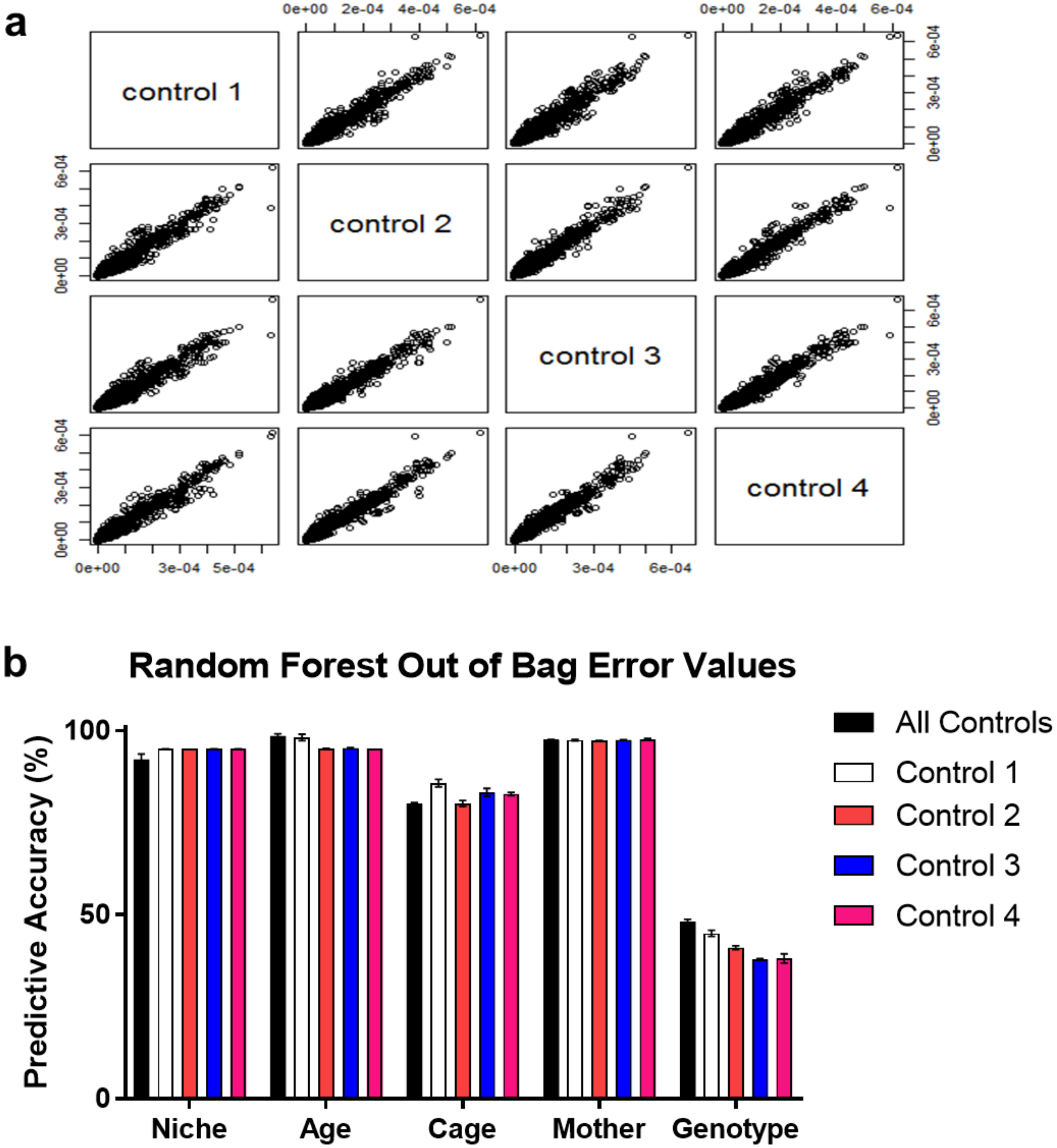

Supplementary Figure S7: Control samples are highly correlated. 16S rRNA was sequenced from the stools and mucus, of 6 and 18 week old, male wildtype (FVB background) and mdr1a-/- mice. Four technical replicate samples were sequenced multiple times to account for variability between sequencing runs. A RF was performed excluding all but one of these control samples and the correlation of 'mean decrease accuracy' values was plotted (a). The predictive accuracy when running a RF forest including all these controls or excluding all but one was plotted (b), for each of the treatment groups (niche, age, cage, mother and genotype). Suppplementary Figure S7a produced in R 3.6.0 for Windows and S7b produced in GraphPad Prism 8.

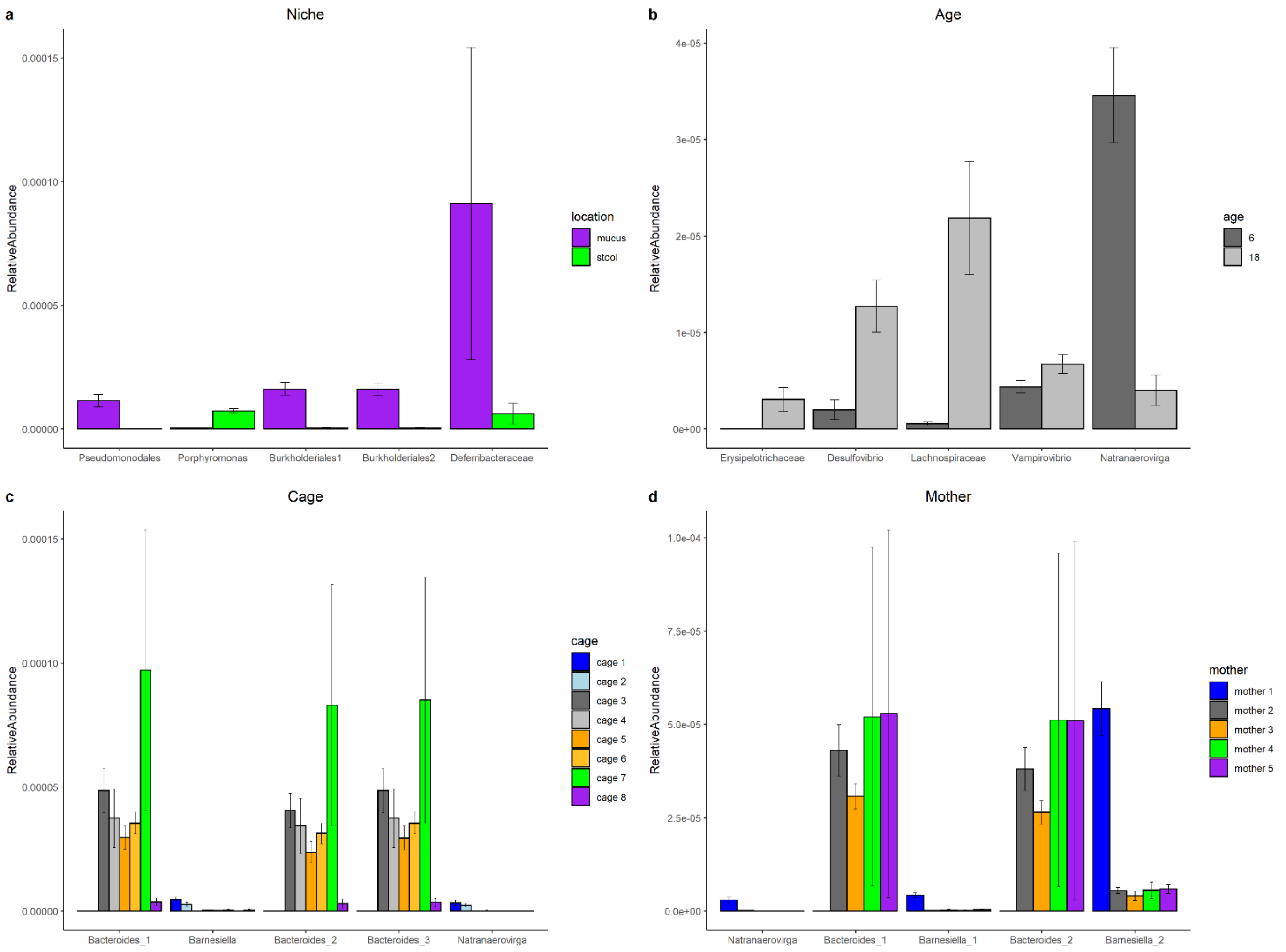

Supplementary Figure S8: Relative abundance of most important clades. A random forest (RF) model was run to find associations between the relative abundance of bacterial clades and different treatment groups: niche (stool vs mucus) (a), age (6 vs 18 week old mice) (b), cage (c) and mother (d). The relative abundance of the five most important clades for each treatment group are shown. Data shown as mean (+/- SEM). Figure produced in R 3.6.0 for Windows.

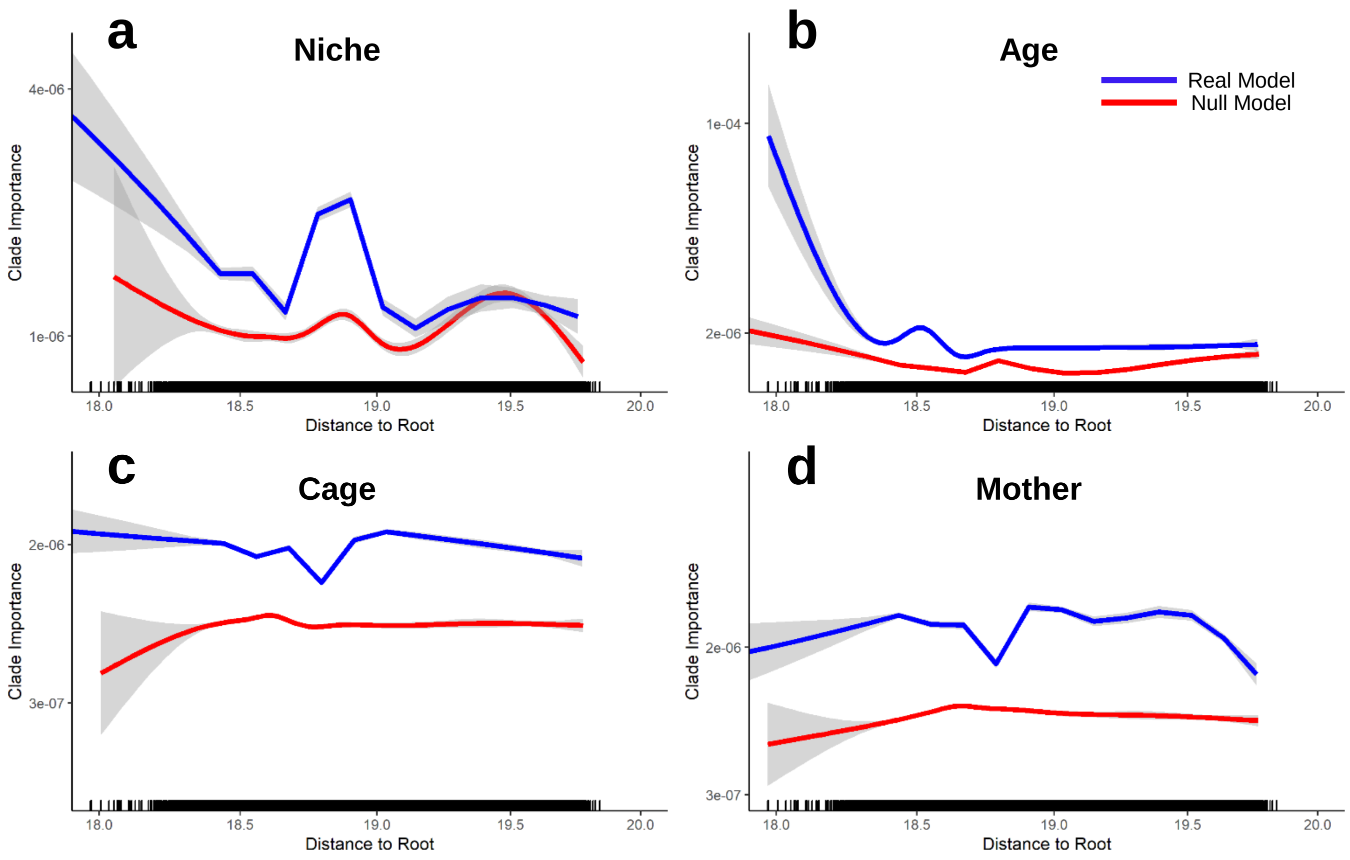

Supplementary Figure S9: Taxa of intermediate level distinguish niche, age and cage. The distance from clade to root was compared against the 'mean decrease accuracy' (MDA) value when running a forest that compared the niche (A), age (B), cage (C) and mother (D). The 'real' random forest model is illustrated in blue and a null (negative control) random forest model is illustrated in red. Figure produced in R 3.6.0 for Windows.

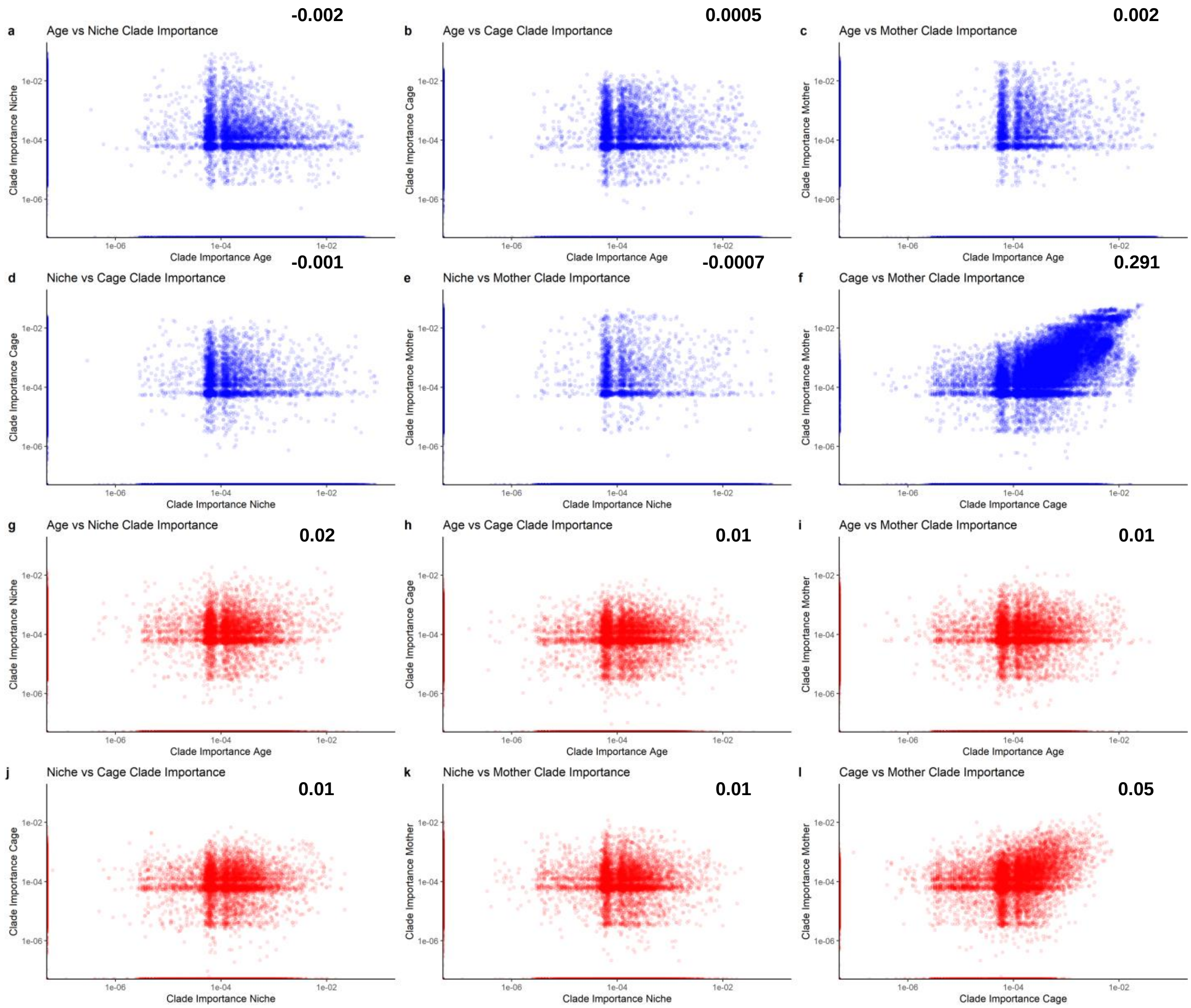

Supplementary Figure S10: Clade importance between forests. The importance (Mean Decrease Accuracy value) of a clade in one forest was plotted against its importance in another forest for the real model (blue): Age vs Niche (a), Age vs Cage (b), Niche vs Cage (c), Age vs Mother (d), Niche vs Mother (e) and Cage vs Mother (f) and the null (negative control, red) model: Age vs Niche (g), Age vs Cage (h), Niche vs Cage (i), Age vs Mother (j), Niche vs Mother (k) and Cage vs Mother (l). Spearman's Rank correlation values are shown for each comparison. Figure produced in R 3.6.0 for Windows.
