# Supplementary material for: Cage and maternal effects on the bacterial communities of the murine gut
Source: Sci Rep. 2021 May 10;11:9841. doi: 10.1038/s41598-021-89185-5 (PMC8110963; doi:10.1038/s41598-021-89185-5)
Supplement: Supplementary file 2 — Supplementary Information. [file 41598_2021_89185_MOESM2_ESM.docx]

**Cage and maternal effects on the bacterial communities of the murine gut**

Gurdeep Singh^1^, Andrew Brass^2^, Sheena M. Cruickshank^1^, and Christopher G. Knight^3^.

^1^ Faculty of Biology, Medicine and Health, Lydia Becker Institute of Immunology and Inflammation, Manchester Academic Health Science Centre, A.V. Hill Building, The University of Manchester, Oxford Road, Manchester, M13 9PT, United Kingdom. [Gurdeep.Singh@manchester.ac.uk](mailto:Gurdeep.Singh@manchester.ac.uk), [Sheena.Cruickshank@manchester.ac.uk](mailto:Sheena.Cruickshank@manchester.ac.uk)

^2^ Faculty of Biology, Medicine and Health, Division of Informatics, Imaging and Data Sciences, Stopford Building, The University of Manchester, Oxford Road, Manchester, M13 9PT, United Kingdom. [Andy.Brass@manchester.ac.uk](mailto:Andy.Brass@manchester.ac.uk)

^3^ Faculty of Science and Engineering, School of Earth and Environmental Sciences, Michael Smith Building, The University of Manchester, Oxford Road, Manchester, M13 9PT, United Kingdom. [Chris.Knight@manchester.ac.uk](mailto:Chris.Knight@manchester.ac.uk)

| **Corresponding author**: |  |
| --- | --- |
| Sheena M. Cruickshank |  |
| A.V. Hill Building |  |
| The University of Manchester |  |
| Oxford Road |  |
| Manchester |  |
| M13 9PT |  |
|  |  |
| Sheena.Cruickshank@manchester.ac.uk |  |
| Phone +44 (0) 161 275 1582 |  |

Running title: **Gut microbiomes of co-housed littermate mice**

**Supplementary Information**

To validate the method for identifying important taxa, we firstly tested the reproducibility of different estimates of clade importance based on the same data. We expect more reproducible estimates (i.e. tighter correlations among estimates) when more trees are used in the random forest, which we find to be true (Supplementary Figure S5a). We also expect there to be a maximum possible correlation among independent importance estimates. By calculating the rank correlation among importance values from independent RFs of different sizes and fitting a saturating function (Supplementary Figure S5b), we estimate that a forest of 100,000 trees, as we use in our analyses, achieves 99% of the maximum correlation of 0.21; i.e. the approach used in the present study gets close to optimal reproducibility. Secondly, we validated the approach to individual importance estimates by taking the most important clade identified for age (within the *Erysipelotrichaceae* family) and redistributing the abundances of this clade to only be present in all WT samples. Upon repeating the random forest separating on genotype, the *Erysipelotrichaceae* clade became the most important and this change meant the genotype RF was now significantly better than the null model (Two Way ANOVA- Sidak’s post hoc test: *P* < 0.05) (Supplementary Figure S6a). Similarly, repeating the random forest separating on age using the redistributed dataset, the *Erysipelotrichaceae* clade ceased to be the most important, leaving most of the other important clades largely unaffected (Supplementary Figure S6b). This suggests that important clades are robust and maintain their importance, even when other clades are altered. Thirdly, we included 4 technical replicates (3 for a single stool sample and 1 for a single mucus sample) that were used as an internal control between sequencing runs, in our forest models. The results of these technical replicates were highly correlated (Supplementary Figure S7a) and which technical replicate was included made little difference to the results (Supplementary Figure S7b).
